# Differential proteomic analysis of children infected with respiratory syncytial virus

**DOI:** 10.1590/1414-431X20209850

**Published:** 2021-02-26

**Authors:** Gen-Quan Yin, Hui-Xuan Zeng, Zi-Long Li, Chen Chen, Jia-Yong Zhong, Mi-Si Xiao, Qiang Zeng, Wen-Hui Jiang, Pei-Qiong Wu, Jie-Min Zeng, Xiao-Yin Hu, Huan-Hui Chen, Ruo-Hu, Hai-Jin Zhao, Lin Gao, Cong Liu, Shao-Xi Cai

**Affiliations:** 1 Chronic Airways Diseases Laboratory, Department of Respiratory and Critical Care Medicine, Nanfang Hospital, Southern Medical University, Guangzhou, Guangdong, China; 2 Guangzhou Women and Children's Medical Center, Guangzhou Medical University, Guangzhou, Guangdong, China; 3 Department of Cardiology, Shenzhen Children's Hospital, Shenzhen, China; 4 Guangdong Food and Drug Vocational College, Guangzhou, Guangdong, China; 5 College of Computer Science, Guangdong Polytechnic Normal University, Guangzhou, Guangdong, China; 6 Pediatric Research Institute, Qilu Children's Hospital of Shandong University, Jinan, Shandong, China; 7 Department of General Practice Medicine, the First Affiliated Hospital, Jinan University, Guangzhou, Guangdong, China

**Keywords:** Children, Respiratory syncytial virus, Mouse model, Differentially expressed protein, Biomarkers

## Abstract

Respiratory syncytial virus (RSV) infection is the main cause of lower respiratory tract infection in children. However, there is no effective treatment for RSV infection. Here, we aimed to identify potential biomarkers to aid in the treatment of RSV infection. Children in the acute and convalescence phases of RSV infection were recruited and proteomic analysis was performed to identify differentially expressed proteins (DEPs). Subsequently, promising candidate proteins were determined by functional enrichment and protein-protein interaction network analysis, and underwent further validation by western blot both in clinical and mouse model samples. Among the 79 DEPs identified in RSV patient samples, 4 proteins (BPGM, TPI1, PRDX2, and CFL1) were confirmed to be significantly upregulated during RSV infection. Functional analysis showed that BPGM and TPI1 were mainly involved in glycolysis, indicating an association between RSV infection and the glycolysis metabolic pathway. Our findings provide insights into the proteomic profile during RSV infection and indicated that BPGM, TPI1, PRDX2, and CFL1 may be potential therapeutic biomarkers or targets for the treatment of RSV infection.

## Introduction

Respiratory syncytial virus (RSV) is a single-stranded RNA virus belonging to the family Paramyxoviridae and the genus *Pneumovirus*. The RSV genome possesses ten genes encoding 11 proteins, including transmembrane surface glycoproteins, proteins that transfer genomic RNA into nucleocapsids, non-structural proteins, transcription and replication factors, and unglycosylated matrix M protein, which play roles in viral attachment, fusion, RSV nucleocapsid release into the cytoplasm, viral transcription and subsequent replication, and in reducing the host antiviral response (1). RSV is the most common cause of lower respiratory infection in infants, with highest morbidity in 2-6-month-old infants. By the time they reach two years old, almost all children have been infected with RSV (2). Although most children display minor or few symptoms, 2-3% of children require hospital treatment for serious respiratory symptoms (3). A long-term prospective cohort study revealed that RSV bronchiolitis was responsible for the augmentation of recurrent asthma later in life (4).

The death of infants infected with RSV primarily occurs in developing countries (5). The general pathological signs of RSV infection have been principally described using biopsies, bronchoalveolar lavage fluid, and autopsy samples derived from severe cases. In acute cases, cytopathological characteristics resulting from viral replication in bronchial epithelial cells have been observed, including intracytoplasmic inclusion bodies and multinucleated giant cells (6). While much is known regarding RSV replication, pathology, and transmission, there remains no efficient method for preventing or curing RSV infection (7). Despite decades of study, no approved RSV vaccine or effective therapeutic agent has emerged; however, there are candidates currently at the preclinical phase or in clinical trials (8).

In the current study, we used proteomics and bioinformatics analyses to detect a novel relationship between RSV infection and the glycolysis pathway, and identify several new potential targets for the treatment of RSV infection.

## Material and Methods

### Study population and sample preparation

Forty children diagnosed with RSV bronchiolitis and treated in Guangzhou Women and Children's Medical Center in February and March 2019 were selected as study subjects, and twenty age- and gender-matched healthy children were selected among the population who went to the health care department for regular physical examination without any symptoms or abnormal physical signs and with normal routine blood test. The children with RSV were divided into acute RSV infection and convalescent. Inclusion criteria were: 1) one month to 2 years old; 2) complete medical history data; 3) symptoms, signs, and related auxiliary examinations matched the diagnosis requirements of “Expert consensus on diagnosis and prevention of bronchiolitis (2014 edition)” (9); and 4) RSV infection verified by PCR using a pharynx swab, nasopharynx suction, or other respiratory tract secretion. Exclusion criteria were: 1) insufficient medical history data; 2) specimen contamination; and 3) immunodeficiency or secondary pulmonary infection such as tuberculosis. All subjects provided 1 mL of nasopharyngeal secretions, which were placed in 2 mL normal saline. RSV antigen was detected by direct immunofluorescence. In addition, peripheral blood was drawn from all children, which was centrifuged at 1000 *g* for 10 min at 4^o^C. Then, serum samples were collected and stored at −80°C. The guardians of all children involved in the study provided written informed consent, and the study was approved by the Ethics Committee of Guangzhou Women and Children's Medical Center [#2015101501], and has been carried out in accordance with The Code of Ethics of the World Medical Association (Declaration of Helsinki).

### Proteomic analysis using isobaric tags for relative and absolute quantitation (iTRAQ)

A pooled sample consisting of equal amounts of each of five experimental samples from patients and healthy children were used to perform iTRAQ analysis according to the manufacturer's instructions (SCIEX, USA) (10). Briefly, prepared serum samples (100 μg protein) were reduced, alkylated, and digested with trypsin (Promega, USA) and then dried and reconstituted in 50 μL 0.5 M triethyl ammonium bicarbonate (TEAB) to produce a mixture of peptide fragments, which were differentially labeled with multiple iTRAQ reagents. The pooled samples from control, RSV convalescence-, and acute-phase groups were labeled with mass 113, 114, and 116 isobaric iTRAQ tags, respectively, in the first set of experiments. For the second set of iTRAQ experiments, each pooled sample was labeled with two different isobaric tags. The control group was labeled with 116 and 117, RSV acute-phase group was labeled with 118 and 121, and RSV convalescence-phase group was labeled with 113 and 114. The labeled samples were finally combined into one sample mixture in each set of iTRAQ experiments and dried with a rotary vacuum concentrator. The peptide mixture was fractionated by high pH reverse-phase HPLC using Gemini-NX C18 column (3 μm, 2×150 mm, 110 A, Phenomenex, USA). Thereafter, the peptides were eluted at a flow rate of 200 μL/min with a gradient of acetonitrile buffer, separated into 20 fractions, and then dried by vacuum centrifugation at 8000 *g* for 4 h at room temperature. NanoLC-MS/MS was carried out using Q Exactive^TM^ (Thermo Fisher Scientific, USA), which was interfaced with an UltiMate 3000 RSLCnano system (Thermo Fisher Scientific). The peptide was dissolved in 0.1% formic acid and 5% acetonitrile and loaded onto a PepMap C18 trapping column (100 μm i.d., 10 cm long, 3 μm resin; Michrom Bioresources, USA) and then separated on the PepMap C18 RP column (2 μm, 75 μm×150 mm, 100 A) at a flow rate of 300 nL/min. The peptides were analyzed by Q Exactive^TM^ (Thermo Fisher). Intact peptides were detected at a resolution of 70,000, and ion fragments were detected at a resolution of 175,000. A data-dependent procedure that alternated between one MS scan followed by 20 MS/MS scans was applied for the top 20 precursor ions above a threshold ion count of 3E6 in the MS survey scan with 40.0 s dynamic exclusion. The electrospray voltage applied was 2.2 kV. Automatic gain control was used to prevent overfilling of the ion trap; 5E4 ions were accumulated for generation of MS/MS spectra. For MS scans, the m/z scan range was 350 to 1800. Fixed first mass was set as 110 m/z. The total run time for each peptide fraction was 65 min. MS results were analyzed using Protein Pilot (version 5.0) and the Paragon Algorithm (SCIEX, USA) for protein database searching. Proteins with confidence coefficients >95% were identified by searching the SWISSPROT database. The preliminarily identified proteins were divided into groups by an enrichment algorithm and compared with proteins in the database. For differentially expressed proteins (DEP) determination, the cutoff value for the fold change in expression between the RSV infection group and the controls was determined through an assessment of technical variation in the experiment. Proteins with the coefficient of variation (CV) <0.5, fold change in expression >2, and <0.05 (Student's *t*-test) between the RSV infection group and the normal control group were considered significant DEP.

### Bioinformatics analysis

The normalized protein fold change between RSV samples and controls are illustrated using a heatmap. Protein classes were identified in the PANTHER database v14.1 (http://pantherdb.org) (11). Gene Ontology (GO) term and Kyoto Encyclopedia of Genes and Genomes (KEGG) pathway enrichment analyses and protein-protein interaction (PPI) network analysis of identified differentially expressed proteins (DEPs) were performed using the online Metascape database (http://metascape.org) (12) and visualized in Cytoscape v3.7.1 (National Institute of General Medical Sciences, USA). The molecular complex detection (MCODE) algorithm was used in Metascape with default settings to identify a densely connected network of PPIs. Reactome pathway analysis of candidate proteins was performed using the Cytoscape plugin ClueGO v2.5.3 (13).

### RSV strain isolation and intranasal RSV challenge in mice

The original RSV strain (serial number 20181224-218) was provided by the Central Laboratory of Guangzhou Women's and Children's Medical Center, and was isolated from the throat swab of a child (male, 2 years old) suffering from bronchopneumonia. HEp-2 cells were cultured in Dulbecco's modified Eagle's medium (DMEM; Gibco, USA) with 10% fetal bovine serum (FBS) to 75-90% confluence. After washing the cells with phosphate-buffered saline (PBS) twice, 500 μL RSV was added and incubated at 35°C with 5% CO_2_ for 2 h. Then, virus maintenance media (DMEM containing 1% FBS and 1% penicillin-streptomycin) was added and cells were incubated at 35°C with 5% CO_2_ for 5 days before assaying for cytopathic effects.

BALB/c mice were purchased from the Experimental Animal Center of Southern Medical University (China) and housed in the specific-pathogen-free environment of the Experimental Animal Center. All animal experiments were approved and conducted according to the guidelines of the Institutional Animal Care and Use Committee of Southern Medical University, and were carried out in accordance with the National Institutes of Health guide for the care and use of laboratory animals (NIH Publications No. 8023, revised 1978). Mice (6-8 weeks old; n=10 each for the control, acute-phase, and convalescence-phase groups) were challenged with RSV. Briefly, each mouse was intranasally inoculated with 10^6^ plaque-forming units of RSV dissolved in 50 µL PBS. At the same time, control mice were intranasally inoculated with 50 µL PBS only. After 3 days, the mice were sacrificed and their lungs were obtained. One portion of each lung tissue was homogenized and stored at −80°C for western blot analysis, and the other was fixed with formalin, dehydrated with ethyl alcohol and xylene, and stored in paraffin for hematoxylin-eosin (HE) staining.

### HE staining

HE staining was conducted as previously described (14). Briefly, mouse lung tissues were sliced into 7-µm sections and attached to glass slides, and then rehydrated with xylene, ethyl alcohol, and water. Sections were stained with hematoxylin for 5 min and sequentially washed with ultra-pure water, 1% hydrochloric acid ethanol, and pure water. Then, sections were stained with 0.5% eosin for 1-3 min, and washed with pure water. After dehydration with ethyl alcohol and xylene and mounting with neutral balsam, sections were observed and imaged under a microscope.

### RNA extraction and real-time PCR

RNA extraction was performed according to the manufacturer's instructions (Takara, China). Briefly, mouse lung samples were homogenized with Trizol, processed with chloroform, isopropanol, and ethyl alcohol, and centrifuged at 12,000 *g* for 10 min at 4°C. Extracted RNA was dissolved in RNase-free double distilled water and reverse transcribed to cDNA using the PrimeScript^TM^ RT kit (Takara, China). Real-time PCR was performed using the SYBR Premix Ex Taq^TM^II kit with RSV primers (forward: 5′-TGCATTGTACCTCACCTCAAGT-3′, reverse: 5′-TTGGCAGCTGGTGTGTTTTG-3′) and actin beta primers (forward: 5′-GAGGTATCCTGACCCTGAAGTA-3′, reverse: 5′-CACACGCAGCTCATTGTAGA-3′) as an internal reference.

### Western blot analysis

Western blot was performed as previously described (15). Briefly, the protein concentrations of the RSV infection serum samples (15 patients with acute- and convalescence-phase RSV infection, 15 control) and mouse lung homogenates (10 mice with acute- and convalescence-phase RSV infection, 10 control) were determined using a BCA kit (Beyotime Biotechnology, China). Protein samples (20 µg) were separated by sodium dodecyl sulfate-polyacrylamide gel electrophoresis at constant voltage in Tris-glycine buffer, and then transferred onto polyvinylidene difluoride membranes at constant current. The membranes were washed thrice in Tris-buffered saline containing 0.1% TWEEN 20 (TBST), blocked with 10% bovine serum albumin for 1 h at room temperature, and incubated with primary antibodies against triosephosphate isomerase 1 (TPI1; ab28760, Abcam, USA), peroxiredoxin 2 (PRDX2; ab109367, Abcam), bisphosphoglycerate mutase (BPGM; ab97497, Abcam), and cofilin 1 (CFL1; ab42824, Abcam) at 4°C overnight. The next day, membranes were washed three times in TBST and incubated with secondary antibody for 1 h at room temperature. After three washes in TBST, protein signals were visualized with ECL (Beyotime Biotechnology, China) and exposed to X-ray film. The band intensities were quantitated using ImageJ software (Version 2.0; NIH, USA) and normalized to GAPDH.

### Statistical analysis

All data are reported as means±SD. Statistical differences for the multiple pairwise comparisons of protein expression levels (acute-phase *vs* control, convalescence-phase vs control) were analyzed using ANOVA with the Dunnett *post hoc* test; P<0.05 was considered significant. All experiments were repeated at least three times.

## Results

### Proteomic analysis of serum from patients with RSV infection

**Figure 1:**
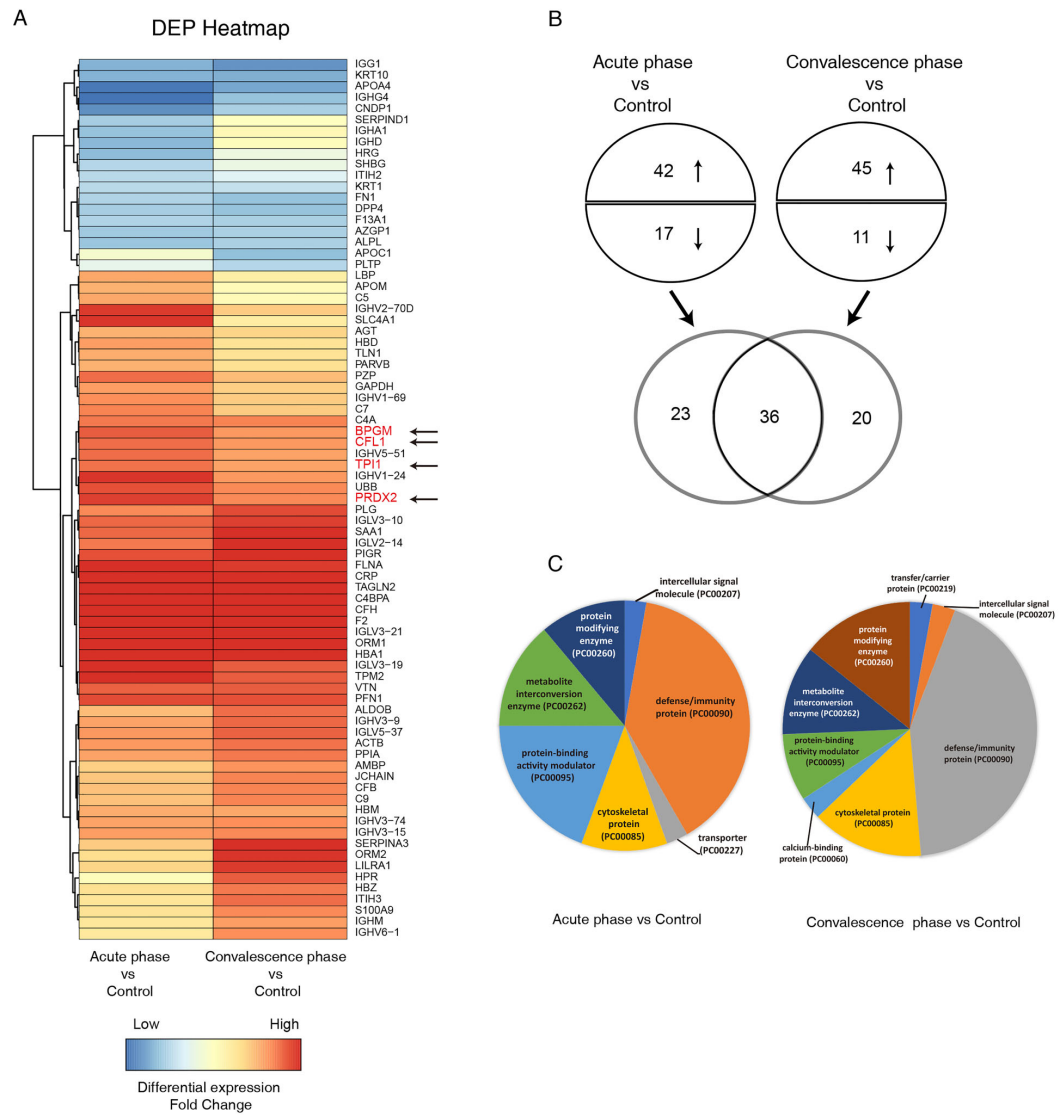
Global profiles, intersection Venn diagram, and protein classification of DEPs between the RSV infection (acute phase and convalescence phase) and control. **A**, Normalized fold change of DEPs of the acute phase of RSV infection group *vs* control group (left column) and the convalescence phase of RSV infection group *vs* control group (right column) is color-coded: red indicates upregulation, and blue indicates downregulation. The arrows indicate the candidate DEPs. **B**, The number of upregulated (red) and downregulated (blue) DEPs from the acute phase and the convalescence phase. The Venn diagram shows the overlapping DEPs. **C**, The protein classes of DEPs in the acute phase (left pie chart) and the convalescence phase (right pie chart) are annotated by PANTHER database. DEP: differentially expressed protein; RSV: respiratory syncytial virus.

Samples from the three groups (control, acute-, and convalescence-phase) were processed and analyzed according to the workflow shown in Figure S1. A total of 463 proteins were identified by comparing expression in the acute- and convalescence-phase RSV infection and healthy controls using iTRAQ (Table S1). The CV was used to evaluate the reproducibility of the pairwise comparison between RSV acute/convalescence phases and control samples. Less than 90% of proteins could be recovered at CV<0.4, while 92∼95% of proteins could be recovered at CV<0.5 (Table S2). For fold change in protein expression, 67∼68% proteins were under 1.5-fold change (fold change ratio between 0.667 and 1.5), 82∼83% proteins were under 2-fold change (fold change ratio between 0.5 and 2), and 88∼90% proteins were under 2.5 fold change (fold change ratio between 0.4 and 2.5). An overly loose cutoff value could increase the false positive of the DEP discovery, while a more stringent cutoff value could reduce the likelihood of true DEP discovery. Thus, the cutoff value of DEP was finalized to be 2-fold change and CV <0.5 with P<0.05. Of these, 79 DEPs between patients with acute-/convalescence-phase RSV infection and healthy controls were identified using a fold change ≥2 as the threshold for upregulation and a fold change ≤0.5 as the threshold for downregulation; their normalized fold change levels are shown in Figure 1A. There were 59 DEPs between acute-phase RSV and control groups, with 42 and 17 proteins upregulated and downregulated, respectively, due to RSV (Figure 1B, Table S3), and 56 DEPs between the convalescence-phase RSV and control groups, with 45 and 11 proteins upregulated and downregulated, respectively (Figure 1B, Table S4). A total of 36 DEPs overlapped between the two RSV infection phases, while 23 DEPs were found only in the acute *vs* control comparison and 20 were found only in the convalescence *vs* control comparison (Figure 1B and Table 1).

**Table 1:** The overlapping differentially expressed proteins (DEPs) in respiratory syncytial virus infection in acute/convalescence groups vs control group.

Overlapping	Count	DEPs
Overlapping DEPs	36	IGG1, APOA4, FN1, IGHG4, DPP4, CNDP1, F13A1, ALPL, AZGP1, HBM, TPI1, IGHV5-51, BPGM, CFL1, IGHV1-24, IGHV3-74, PRDX2, IGHV3-15, UBB, C4A, PPIA, ACTB, IGHV3-9, IGLV5-37, PFN1, PLG, IGLV3-10, FLNA, IGLV2-14, PIGR, HBA1, IGLV3-21, F2, C4BPA, ORM1, CRP
DEPs in acute *vs* control group only	23	KRT10, KRT1, ITIH2, SHBG, HRG, SERPIND1, IGHD, C5, APOM, IGHA1, LBP, SLC4A1, HBD, TLN1, PARVB, AGT, GAPDH, IGHV1-69, IGHV2-70D, C7,PZP, VTN, SAA1
DEPs in convalescence *vs* control group only	20	APOC1, PLTP, IGHM, AMBP, IGHV6-1,S100A9, JCHAIN, C9, CFB, HBZ, ITIH3, ALDOB, TPM2, HPR, IGLV3-19, LILRA1, ORM2, CFH, SERPINA3, TAGLN2

### Functional analysis of DEPs

Protein classes represented by the DEPs were characterized using the PANTHER database. The major protein classes represented among DEPs when comparing acute with control groups (Figure 1C, Table S5) were defense/immunity protein (38.9%), protein-binding activity modulator (19.4%), metabolite interconversion enzyme (13.9%), protein modifying enzyme (11.1%), and cytoskeletal protein (11.1%). The protein classes that were represented the most when we compared the convalescence group with the control group (Figure 1C, Table S5) were defense/immunity protein (42.9%), cytoskeletal protein (14.3 %), protein modifying enzyme (14.3%), metabolite interconversion enzyme (11.4%), and protein-binding activity modulator (8.6%).

**Figure 2:**
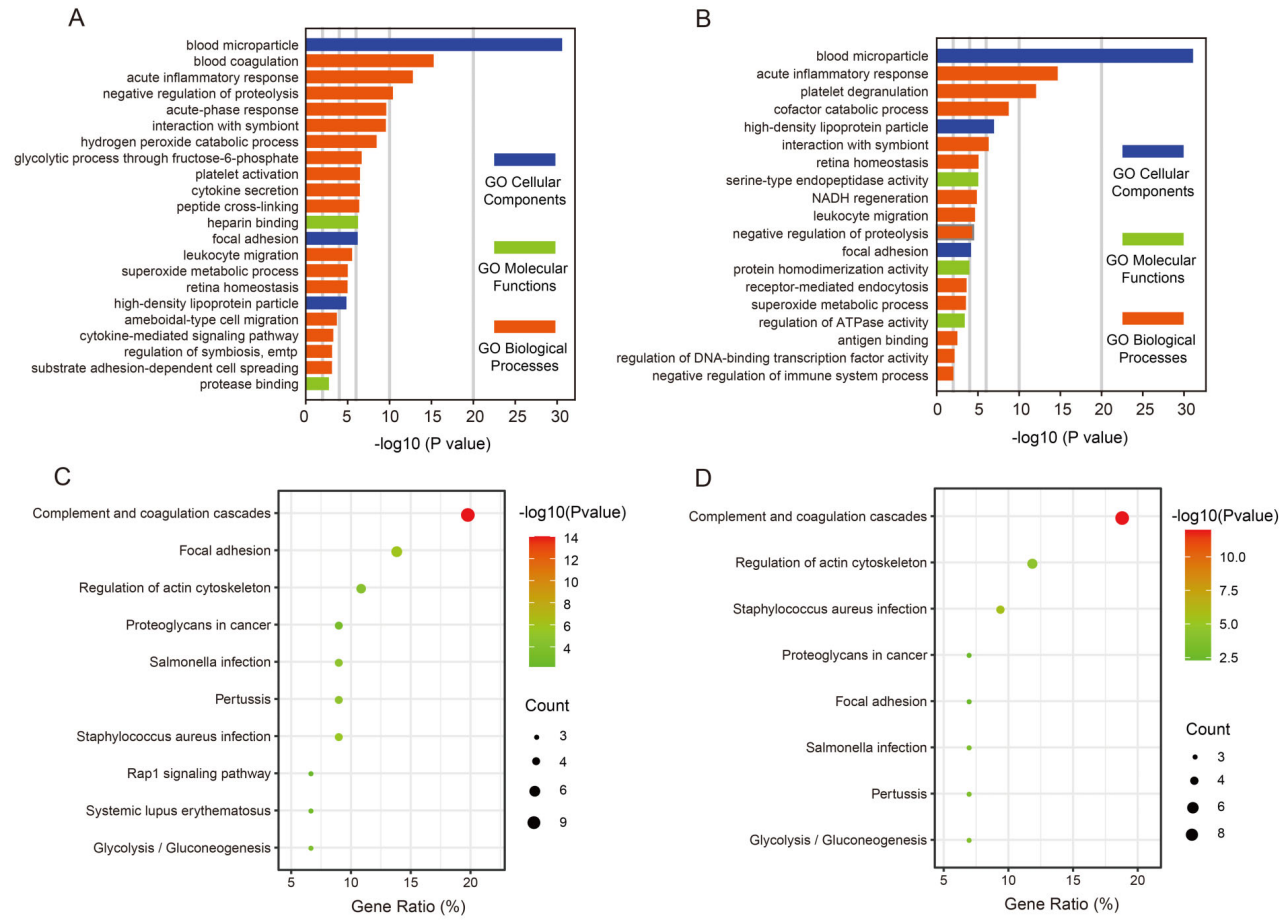
GO and KEGG enrichment analyses of DEPs from acute phase of RSV infection (**A**, **C**) and convalescence phase of RSV infection (**B**, **D**). **A** and **B**, The significantly enriched GO terms (P<0.05) are shown in a negative log10 scale of P values. **C** and **D**, Scatter plot of enriched KEGG pathways (P<0.05) for DEPs. Color and size of dots represent probability of the adjusted P value and the number of matched DEPs, respectively. The X-axis represents the proportion of DEPs in each pathway category. DEPs: differentially expressed proteins; RSV: respiratory syncytial virus; GO: gene ontology; KEGG: Kyoto Encyclopedia of Genes and Genomes.

Enrichment analyses were performed to identify GO terms and KEGG pathways that were significantly associated with DEPs resulting from acute- and convalescence-phase RSV infection. Enriched cellular component, molecular function, and biological process GO terms are presented in Figure 2. Among the DEPs from the acute *vs* control comparison (Figure 2A) and the convalescence *vs* control comparison (Figure 2B), significantly enriched cellular component terms were blood microparticle, focal adhesion, and high-density lipoprotein particle. For molecular functions, DEPs were enriched with proteins involved in heparin binding, protease binding, serine-type endopeptidase activity, protein homodimerization activity, and regulation of ATPase activity. For biological processes, DEPs were enriched with acute inflammatory response, platelet degranulation, acute-phase response, antioxidant activity, and glycolytic process through fructose-6-phosphate. Significantly enriched KEGG pathways involving DEPs included complement and coagulation cascades, *Staphylococcus aureus* infection, cholesterol metabolism, pertussis, focal adhesion, and *Salmonella* infection (Figure 2C and D), which are commonly enriched pathways in the presence of other infectious diseases. Glycolysis/Gluconeogenesis pathway was also included among significantly enriched KEGG pathways.

### PPI network analysis and hub protein identification

**Figure 3:**
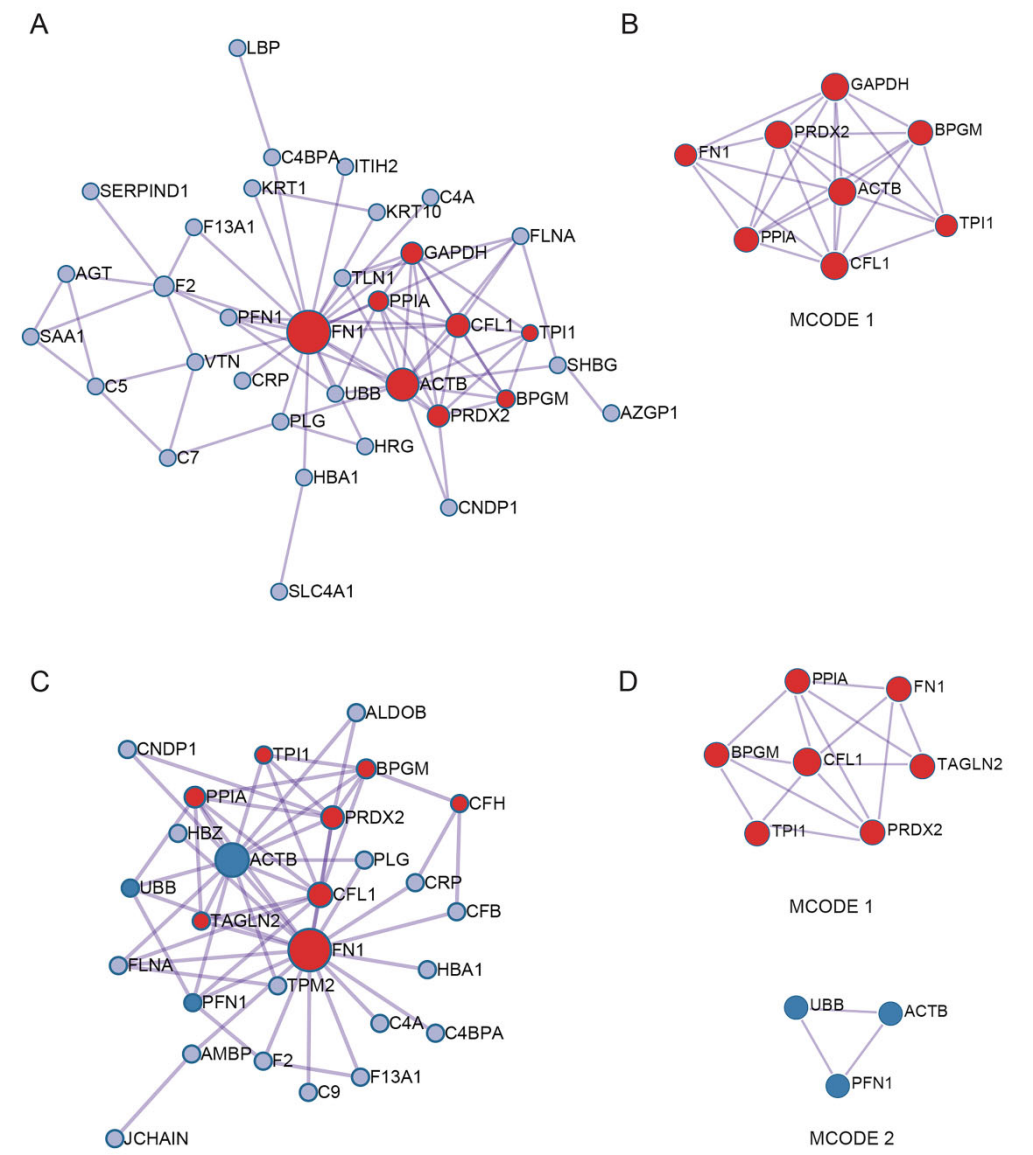
Protein-protein interaction (PPI) network and identification of MCODE components of the DEPs. **A** and **C**, PPI network of DEPs from the acute phase of RSV infection (**A**) and convalescence phase (**C**). **B** and **D**, MCODE identification in the PPI network. DEP: differentially expressed protein; RSV: respiratory syncytial virus; MCODE: Molecular Complex Detection.

To examine the possibility of interactions between DEPs and identify important hub proteins among them, PPI networks were constructed and MCODE complexes were identified. The full PPI network of the 79 DEPs and the MCODE complexes from both comparisons are shown in Supplementary Figure S2 and details are provided in Table 2. The represented functional GO/KEGG enrichment terms of these three MCODE complexes demonstrate their association with canonical glycolysis, NADH regeneration, membrane attack complex, and actin filament binding. Complexes composed of DEPs from the acute *vs* control and convalescence *vs* control comparisons are shown in Figure 3A and C, respectively, and the significantly represented functional enriched GO terms and KEGG pathways of hub proteins in each complex (acute *vs* control shown in Figure 3B, convalescence *vs* control shown in Figure 3D) are listed in Table 2 and Table S6. Hub proteins in the acute *vs* control comparison were associated with GO terms such as canonical glycolysis, glucose catabolic process to pyruvate, and NADH regeneration, while hub proteins in the convalescence *vs* control comparison were associated with the terms isomerase activity, regulation of multi-organism process, and cofactor metabolic process (Table 2 and Table S6).

**Table 2:** Molecular complex detections (MCODEs) functional enrichment analysis - acute and convalescence phases of respiratory syncytial virus (RSV) infection.

	MCODEs	Hub Genes	Represented enriched GO terms
Acute phase *vs* control	MCODE1	BPGM, TPI1, PPIA, CFL1, PRDX2, ACTB, GAPDH, FN1	canonical glycolysis (GO:0061621)glucose catabolic process to pyruvate (GO:0061718)NADH regeneration (GO:0006735)
Convalescence phase *vs* control	MCODE1	BPGM, TPI1, PPIA, CFL1, PRDX2, GAPDH, FN1	isomerase activity (GO:0016853)regulation of multi-organism process (GO:0043900)cofactor metabolic process (GO:0051186)
	MCODE2	UBB, PFN1, ACTB	
RSV infection *vs* control	MCODE1	BPGM, TPI1, PPIA, CFL1, PRDX2, ACTB, GAPDH, FN1	canonical glycolysis (GO:0061621)NADH regeneration (GO:0006735)glucose catabolic process to pyruvate (GO:0061718)
	MCODE2	C5, C7, C9, VTN	membrane attack complex (GO:0005579)regulation of complement activation (GO:0030449)regulation of protein activation cascade (GO:2000257)
	MCODE3	TLN1, TPM2, FLNA	actin filame0nt binding (GO:0051015)muscle contraction (GO:0006936)actin binding (GO:0003779)

**Figure 4:**
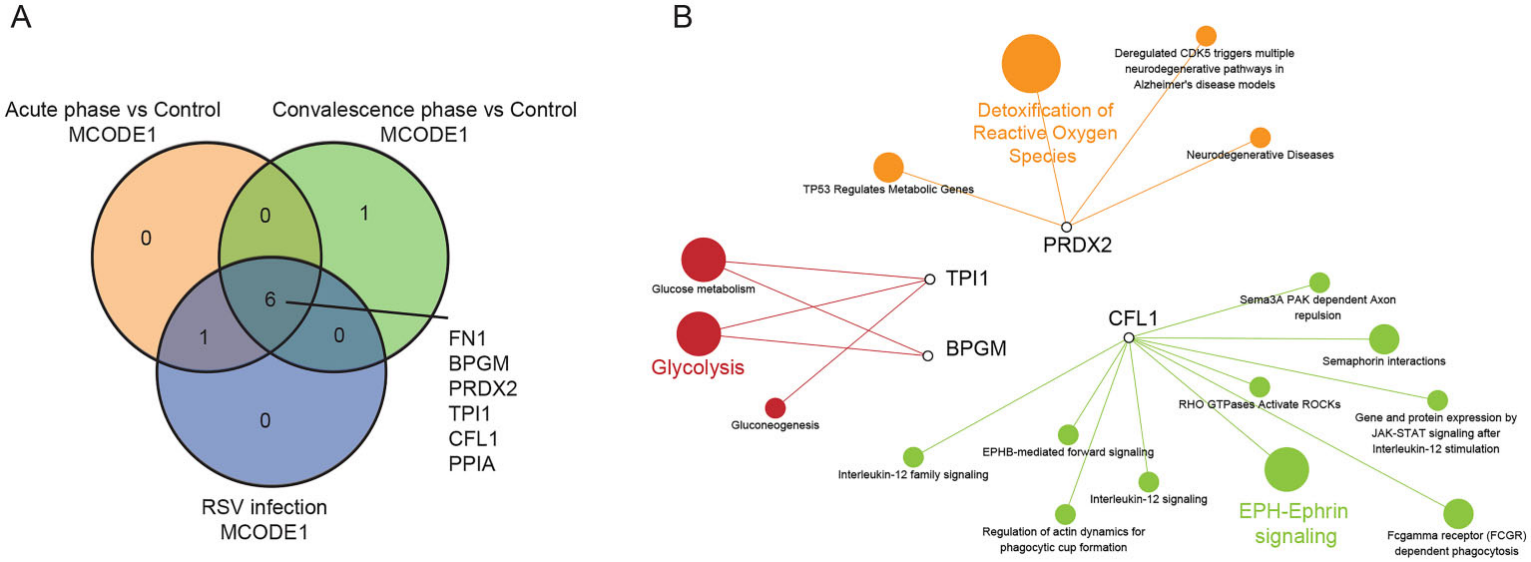
Candidate protein selection and pathway annotation. **A**, The Venn diagram of MCODE1 hub proteins from RSV infection of acute phase, convalescence phase, and the union DEPs of the two phases (ALL-DEPs). **B**, The Reactome pathway annotation of candidate proteins. DEP, differentially expressed protein; RSV: respiratory syncytial virus; MCODE: Molecular Complex Detection.

Interestingly, several hub proteins overlapped between the comparisons. Six hub proteins (BPGM,TPI1, PPIA, CFL1, PRDX2, FN1) overlapped between the MCODE1 complexes formed based on the acute *vs* control, convalescence *vs* control, and the merged DEP comparison (Figure 4A). These hub proteins were upregulated in the RSV infection except FN1. The fold change in the expression of PPIA between convalescence and control groups was larger than that in acute *vs* control comparison, which was different from the other four DEPs. The fold changes in expression of PRDX2, TPI1, BPGM, and CFL1 in acute-phase *vs* control were larger than those in convalescence phase *vs* control comparison. Hence, we performed further analysis on PRDX2, TPI1, BPGM, and CFL1. Reactome pathway annotation of PRDX2, TPI1, BPGM, and CFL1 was performed (Figure 4B). BPGM and TPI1 were mainly associated with the glycolysis pathway; PRDX2 was associated with pathways regulating the detoxification of reactive oxygen species (ROS), and CFL1 was associated with the ephrin signaling pathway. Based on these results, PRDX2, TPI1, BPGM, and CFL1 were selected as candidate proteins for further validation.

### Validation of candidate proteins in clinical samples by western blotting

**Figure 5:**
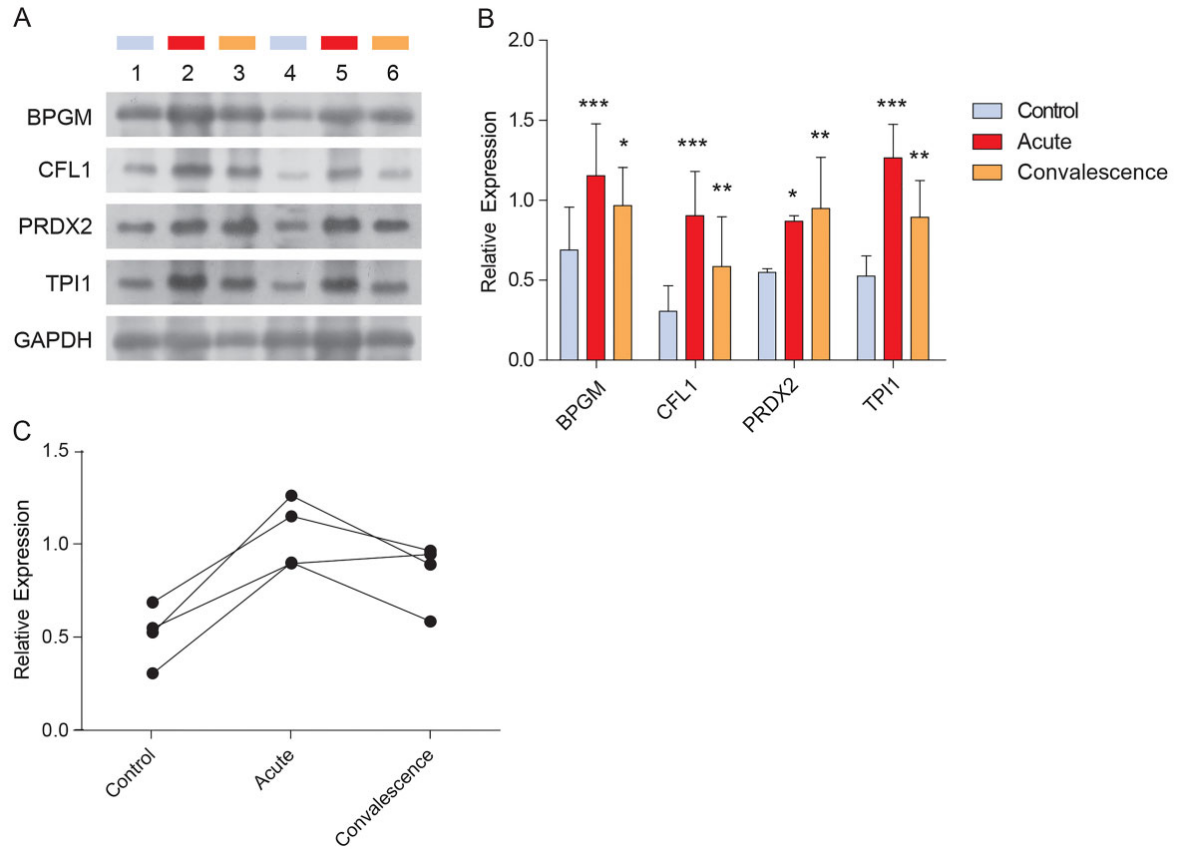
A, Western blot validation of four candidate proteins (BPGM CFL1, PRDX2, TPI1) from acute phase of RSV infection, convalescence phase, and control samples. Each group included 10 biological replicates and GAPDH was used as an internal control. **B**, Analysis of the western blot band intensities of candidate proteins in panel **A**, and comparison of expression levels between RSV infection and control groups. *P<0.05, **P<0.01, ***P<0.001 compared to control (ANOVA with the Dunnett *post hoc* test). **C**, Trends in expression levels of candidate proteins in the three groups. Data are reported as means±SD of 10 biological replicates. RSV: respiratory syncytial virus.

To validate the iTRAQ results, the expression levels of BPGM, CFL1, PRDX2, and TPI1 in clinical samples were examined by western blotting (Figure 5). The expression levels of all four proteins were significantly upregulated in both the acute and convalescence phases compared to the controls (Figure 5A and B). The highest expression levels of the four candidate proteins were observed in the acute-phase group, with relatively decreased expression in the convalescence-phase group (Figure 5B and C). These results were highly consistent with the proteomic quantification results.

### Validation of candidate proteins in a mouse model of RSV infection

**Figure 6:**
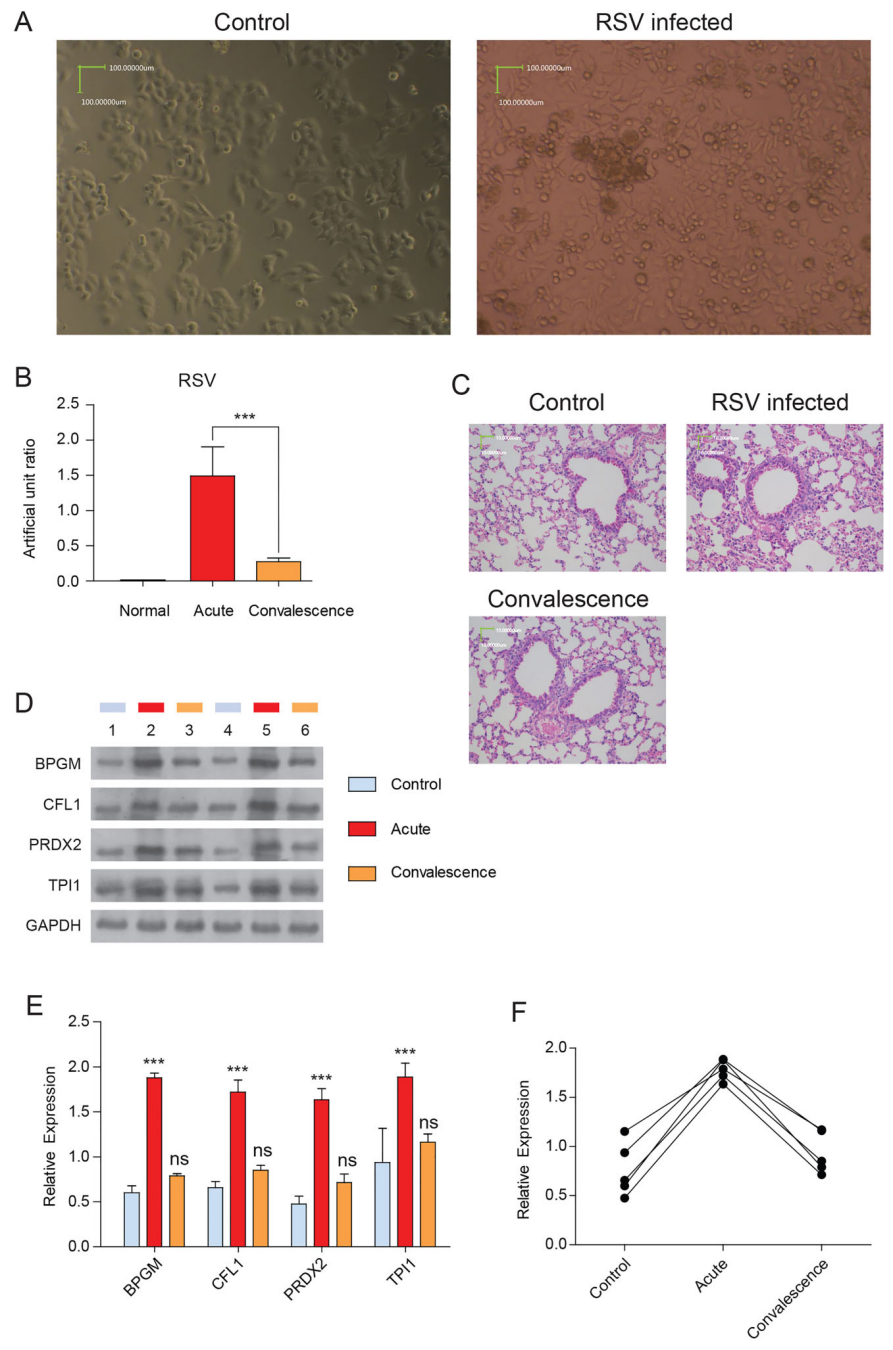
Pathological changes in lung tissues and validation of candidate DEPs in RSV-infected mice models. **A**, Hep-2 cells displayed obvious cytopathic effect after RSV infection. Control: Hep-2 cells. RSV infected: Hep-2 cells infected with RSV 5 days later (scale bar, 100 μm). **B**, Real-time qPCR quantification of RSV-specific gene expression in mice lung tissue of control, RSV acute infected, and convalescence group. Data are reported as means±SD of 10 biological replicates. **C**, Pathological changes in lung tissues (stained with HE) (scale bar, 10 μm). Control: lung tissues of control mice; RSV infected: lung tissues of RSV infected mice; Convalescence: lung tissue of mice during the recovery period. **D**, Western blot validation of four candidate proteins (BPGM, CFL1, PRDX2, TPI1) in samples from the three groups; each group included three biological replicates and GAPDH was used as an internal control. **E**, Comparison analysis of the western blot band intensities of RSV infection and control group (acute phase *vs* control and convalescence phase *vs* control). **F**, Trends in expression levels of candidate proteins in the three groups. Data are reported as means±SD of 10 biological replicates. The multiple comparisons test of acute phase *vs* control and convalescence phase *vs* control were performed using ANOVA with Dunnett *t*-test. ***P<0.001 *vs* control. ns: not significant; DEP: differentially expressed protein; RSV: respiratory syncytial virus; HE: hematoxylin and eosin.

We next investigated the expression levels of BPGM, CFL1, PRDX2, and TPI1 in RSV-infected mice. Five days after RSV infection, HEp-2 cells displayed significant cytopathic effect (Figure 6A). Next, the mice underwent an intranasal challenge with RSV. The RSV artificial unit ratios displayed significant changes at every stage of infection (Figure 6B). The histopathology of control mouse lung tissues showed no inflammatory cell infection in the alveolar wall, and the pulmonary alveoli were neat and clean, indicating no inflammation in the control group (Figure 6C). In contrast, the lung tissues of RSV-infected mice showed thickened alveolar walls, with infiltration of inflammatory cells (mononuclear cells, lymphocytes), demonstrating obvious inflammation in the acute-phase group (Figure 6C). Notably, the inflammatory cells in the alveolar wall of the lung tissues of infected mice in the convalescence phase were decreased (Figure 6C). These results indicated that a mouse model of RSV infection was successfully established. RSV levels were below the range of detection in the control group, were positive and highly expressed in the acute-phase group, and positive but showed lower expression in the convalescence-phase group. In addition, the expression levels of BPGM, CFL1, PRDX2, and TPI1 were all significantly upregulated in RSV-infected mice (acute group) compared to those in the control group, and marginally but not significantly upregulated in the convalescence group compared to those in the control group (Figure 6D, E, and F). These trends were consistent with those observed by the iTRAQ-based quantification and western blot analysis.

## Discussion

RSV infection results in millions of hospital admissions and thousands of in-hospital deaths in children younger than five years of age every year (16). There is an urgent need to identify novel biomarkers related to the pathophysiological changes occurring during disease progression to improve the current treatment options and disease prognosis. In this study, we identified DEPs between children with acute- and convalescence-phase RSV infections and healthy controls by iTRAQ-based quantitative proteomics and through validation study confirmed four DEPs, namely BPGM, CFL1, PRDX2, TPI1, to be significantly upregulated in RSV infection. Interestingly, functional analysis indicated that two of the candidate proteins, BPGM and TPI1, are mainly involved in glycolysis. Thus, we report an association between RSV infection and the glycolysis metabolic pathway-related proteins BPGM and TPI1, which we propose may serve as novel potential therapeutic targets for RSV infection.

BPGM is a glycolytic enzyme that converts 1,3-diphosphoglycerate to 2,3-diphosphoglycerate (2,3-DPG), a highly expressed small molecule in red blood cells that regulates the oxygen affinity of hemoglobin. Therefore, BPGM can impact erythrocyte function by regulating the level of 2,3-DPG. BPGM has been reported to be expressed only in erythrocytes and placental cells (17), but mRNA and protein expression data from the Human Protein Atlas indicate its expression in multiple human tissues (https://www.proteinatlas.org/ENSG00000172331-BPGM/tissue) (18). BPGM has been linked to diseases involving erythrocyte abnormalities, such as secondary congenital erythrocytosis (19) and erythrocytosis (20); however, little is known about the link between BPGM and RSV infection. A previous study reported that gene expression of BPGM is significantly upregulated in infants with RSV bronchiolitis (21). In our study, we found that the protein expression level of BPGM was also associated with RSV infection.

TPI1 is a glycolytic enzyme that catalyzes the isomerization of glyceraldehyde 3-phosphate (G3P) and dihydroxyacetone phosphate (DHAP) to produce critical energy for cells (22). In addition to glycolysis, TPI1 is also involved in glucose metabolism and gluconeogenesis. Deregulation of TPI1 impairs glycolysis, decreasing the cell's energy supply. Mutations in TPI1 can cause triosephosphate isomerase deficiency, which reduces the immune activity of white blood cells and increases the risk of viral infection. An association between TPI1 expression and RSV infection has not been previously reported, but several studies have observed close relationships between viral infections and energy metabolism pathways. Zhang et al. (23) found associations between saccharide and polysaccharide metabolism-related pathways and H7N9 infection. In addition to affecting energy metabolism, TPI1 converts DHAP into G3P, avoiding the cytotoxicity caused by excessive DHAP accumulation in erythrocytes. In this study, we found that BPGM and TPI1 were upregulated in RSV-infected samples, and validated this trend in clinical samples and in mice. Recent studies have demonstrated that the cellular regulation of glycolytic metabolism is a crucial component of antiviral defense (24–26). However, the exact roles of BPGM and TPI1 in the regulation of RSV infection will require further study.

CFL1, a member of the actin-depolymerizing factor/cofilin protein family, is a widely distributed intracellular actin-modulating protein that controls the polymerization and depolymerization of F- and G-actin in a pH-dependent manner. CFL1 participates in the regulation of cell morphology and cytoskeletal organization, and is involved in the translocation of actin-cofilin complexes from the cytoplasm to the nucleus. While it is a biomarker for cancer (27,28), prior to this study, an association between CFL1 expression and RSV infection has not been reported.

PRDX2 is a member of the peroxiredoxin family of antioxidant enzymes, which catalyze the reduction of hydrogen peroxide and alkyl hydroperoxides. In addition to providing antioxidant protection, PRDX2 may also bolster CD8+ T-cells against viral infection (29,30). According to the Reactome database, PRDX2 is related to pathways involved in ROS detoxification, Alzheimer's disease, and neurodegenerative diseases. RSV infection is associated with significant decreases in superoxide dismutases 1 and 3, catalase, and glutathione S transferase expression *in vivo*, leading to cellular ROS accumulation (31). Endogenous ROS act as signaling molecules, activating the immune system during viral infections (32). However, when ROS production exceeds the capacity of cellular antioxidant defenses, oxidative damage occurs. Regulatory mechanisms exist that precisely manipulate ROS levels, controlling the balance between signaling and damage. PRDX2 is involved in ROS regulation in *Caenorhabditis elegans*, with beneficial effects on neuronal function (33). Not just a simple antioxidant enzyme, PRDX2 also has redox signaling and chaperone functions that are involved in the regulation of stress resistance in many model organisms, including mice, *C. elegans*, and *Drosophila melanogaster* (34). Regulation of PRDX2 to manipulate ROS levels and activate the immune system may be an important strategy against RSV infection. Though an association between PRDX2 and RSV infection has been previously reported (35), no study has explicitly suggested that PRDX2 could be used as a biomarker or therapeutic target for RSV infection. Previous studies and the results described here suggest that PRDX2 plays an important role in the physiological regulation of RSV infection and may be a promising candidate therapeutic target.

Our results suggest that the four proteins described above may have important effects on antiviral regulatory processes, and their roles in RSV infection are worthy of further study. In addition, the HSP90 family members HSP90AA1 and HSP90AB1 were also identified as hub DEPs. HSP90 proteins are molecular chaperones that are highly expressed in various tissues and cell types and play essential roles in many cellular processes (36). As many studies have demonstrated that HSP90 family proteins can be used as antiviral biomarkers and therapeutic targets, including for RSV (36,37), we will perform further experiments on these proteins in future research.

The main limitation of the current study was its small sample size, leading to low statistical power when identifying DEPs in the iTRAQ proteome analysis. In addition, clinical samples were taken from only one hospital, and the robustness of the candidate proteins will need further validation in a larger cohort. For the iTRAQ experiments, we pooled 5 samples together for each group, as is conventionally done to avoid skewing of the results due to outliers from single samples. However, since only one pooled sample was analyzed in each group, random errors could increase noise in iTRAQ proteome results and lower the reproducibility. Validation was performed only for BPGM, CFL1, PRDX2, and TPI1, while the accuracy of the other identified DEPs requires validation in further studies. Finally, although this study identified four proteins to be significantly associated with RSV infection in children, their roles in the pathogenesis of the disease remain unknown.

In summary, we profiled DEPs in the peripheral blood of children infected by RSV and identified four potential therapeutic targets for RSV infection. In future studies, we will focus on verifying the consistency of the levels of these four candidate proteins in a larger RSV infection clinical cohort and elucidating the functional regulation of these proteins in RSV infection.

## Supplementary Material

Click here to view [pdf].
